# Effectiveness of a Multi-Strategy Behavioral Intervention to Increase Vegetable Sales in Primary School Canteens: A Randomized Controlled Trial

**DOI:** 10.3390/nu14194218

**Published:** 2022-10-10

**Authors:** Astrid A. M. Poelman, Shadia Djakovic, Jessica E. Heffernan, Maeva Cochet-Broch, Rebecca K. Golley, David N. Cox, Janne Beelen

**Affiliations:** 1 CSIRO Health & Biosecurity, Westmead, NSW 2145, Australia; 2 Healthy Kids Association, St Leonards, NSW 2065, Australia; 3 CSIRO Agriculture & Food, North Ryde, NSW 2113, Australia; 4 Caring Futures Institute, College of Nursing and Health Sciences, Flinders University, Bedford Park, SA 5042, Australia; 5 CSIRO Health & Biosecurity, Adelaide, SA 5000, Australia

**Keywords:** children, vegetable intake, primary school canteens, behavioral intervention, provisioning, choice architecture, RCT, sales, vegetable waste

## Abstract

Children’s vegetable intake remains inadequate and school canteens may provide opportunities to address this public health concern. This study aimed to determine the effectiveness of an 8-week multi-strategy behavioral intervention that included vegetable provisioning and online menu architecture on vegetable sales in primary school canteens. A randomized controlled trial was undertaken in 16 Australian primary schools (*n* = 4302 students). The control arm kept their regular canteen menu. The primary outcome was vegetable sales measured by assessing vegetable content (in grams) from all menu items and using canteen sales (ordered online and over-the-counter) to calculate vegetable sales (in grams/week) at baseline (3 weeks) and during intervention implementation (8 weeks). Secondary outcomes were vegetable sales in subcategories, intervention acceptability among canteen managers and vegetable waste (four schools). Linear mixed model analysis showed that from baseline to follow-up, the intervention group had significantly higher weekly vegetable sales overall compared with the control group (2707 g/week, 95% CI 1276 to 4137 g/week; *p* < 0.001), with increased vegetable sales in the subcategories of burgers, hot foods and snacks, but not in sandwiches and pasta/rice dishes. The intervention did not lead to more vegetable waste, nor to a decrease in canteen revenue. The canteen managers found the intervention easy to implement and felt children responded favorably to three of the seven strategies. In conclusion, a multi-strategy behavioral canteen intervention increased vegetable sales amongst primary school students.

## 1. Introduction

Consumption of a diet high in fruit and vegetables reduces the risk of chronic diseases, including cardiovascular disease and cancer [1,2,3]. However, in most countries, children do not meet dietary guidelines for vegetable intake [4,5]. The recommended vegetable intake for Australian primary-school-aged children is 4.5–5 serves/day [6], but the average intake of this age group is less than 2 serves/day [6]. Less than 4% of primary-school-aged children meet the recommended vegetable intake [7].

The school food environment could be an opportune setting for facilitating students’ increased vegetable intake through school canteens. Australian schools function as a ‘tuck shop’, a small shop where students can buy a range of lunch, snack and drink items [8]. Currently, vegetable provisioning in Australian schools is low and school canteen nutrition guidelines do not quantify the presence of vegetables in meals or snacks [9,10]. A systematic analysis of 112 Australian online school canteen menus found an average menu to contain 80 items, with 30% containing vegetables. Most sandwiches (60%) and hot foods (54%) did not contain any vegetables [11]. Hence, vegetable intakes at school are low. Whereas Australian children consume 39% of daily dietary energy at school, only 13% of vegetable intake was consumed at school [12]. In the UK, vegetable intake at school similarly contributed to 11% of vegetable intake only [13]. In contrast, US children taking part in the National School Breakfast and School Lunch Program (NSLP) were provided with 47% of their daily energy and 41% of their vegetable intake through these two programs, with vegetable intake deriving solely from the NSLP [14]. A recent analysis of the most recent Australian national dietary survey (NNPAS2011-13) data further found that 83% of Australian children consumed no serves of vegetables during school hours on the day of the survey [7]. Furthermore, there was no difference in the amount of vegetables consumed at school in children who purchased or did not purchase from the canteen [12], whereas US children taking part in the NSLP were more likely to have vegetables than children with a lunch brought from home (29 vs. 13%) [15].

Systematic reviews and meta-analyses show that school-based fruit and vegetable interventions increased fruit consumption but had minimal impact on vegetable intake [16,17]. Thus, novel and more vegetable-specific interventions are needed to increase children’s vegetable consumption in schools. Interventions in Australian school canteens have focused on increasing nutritional quality [18] or fruit and vegetable positioning [19], and to our knowledge, vegetable-specific interventions are lacking.

Several strategies can be employed to increase vegetable offerings and demand, notably food provisioning and behavioral strategies. Food provisioning strategies can include increasing the vegetable content in existing (popular) dishes or introducing new dishes with a high vegetable content [11]. It is essential that altered and new dishes take children’s sensory preferences into account, as their food choices are driven by sensory acceptance [11,20]. A new evidence-based model for development of vegetable-based products has recently been proposed, the Children’s Acceptance Model for Product Development of Vegetables (CAMPOV model) [20]. This model considers sensory (e.g., bright colors, taste, bite-sized), extrinsic (e.g., characters, sensory claims) and psychological (e.g., fun, associative learning, previous experience) factors to increase children’s acceptance of vegetable-based products [20]. This model was derived from evidence that sensory properties of vegetables do not align well with innate likes (sweet taste) and early-in-life established preferences (salty taste, fatty mouthfeel) [21], and as a consequence, vegetable liking is largely a learned behavior [22,23]. Secondly, behavioral strategies can be deployed that are less contingent on explicit decision making. Nudging, or choice architecture, refers to changing the environment in which decisions are made to alter behavior, without forbidding any options or significantly changing monetary incentives of choices [24,25,26,27]. One such strategy involves the positioning of items physically or listing in menus first (primacy) or last (recency). A systematic review found that physical positioning affected food choice amongst adults and children [26]. In online settings, the “primacy” effect is of relevance, a tendency to pay more attention to the first item in a list [28]. So far, positioning in online menu ordering has found mixed results. A behavioral online menu architecture program that included positioning changes successfully lowered energy content of lunch orders of Australian primary school students [18], but positioning of fruit and vegetable snacks as separate first (primacy) and last (recency) categories in online primary school canteen menus did not increase the selection of those snacks [19]. Another nudging strategy is a change in the default option, for example adding a vegetable as the default and allowing the student to omit it (“opt-out” rather than “opt-in”). This taps into the groundbreaking work of Nobel Prize winners Kahneman and Tversky, through challenging rational consumer decision making [29]. Prospect theory predicts that the emotional impact of a loss (loss aversion) far outweighs positive impacts of a potential gain. Therefore, a change in reference level or status quo (i.e., the addition of an item) has been shown to lead to altered choices and even reversals of preference [29]. To date, food applications utilizing prospect theory are scarce, and as far as we are aware, have not been evaluated in a school canteen environment. This study aimed to determine the effectiveness of an 8-week multi-strategy behavioral intervention that included vegetable provisioning and changes to online menu architecture in primary school canteens on vegetable sales. The multiple strategies used were: increasing the vegetable content in existing offerings, adding new vegetable containing food items, using an opt-out rather than opt-in strategy for vegetables as an online menu architecture change and primacy of listing vegetables first as an online menu architecture change. Further, practical support was offered to canteen managers and changes were communicated to students and parents. The primary outcome was vegetable sales (in g/week) from online and over-the-counter canteen sales.

Secondary outcomes were:

- Canteen vegetable sales in subcategories of school menus (burgers, hot meals, other hot foods, sandwiches/wraps/rolls, and snacks);
- Vegetable waste (to validate the assumption that sales are a good proxy for intake) and canteen sales revenue (as a measure of potential adverse effect);
- Acceptability of intervention strategies and implementation by canteen managers.

It was hypothesized that a multi-strategy behavioral intervention would increase vegetable sales when compared with canteens that continued to use their regular menu.

## 2. Materials and Methods

A randomized controlled trial (RCT) with two parallel arms with equal allocation rate was conducted. The intervention arm changed the vegetable offering and menu architecture in canteen menus during an 8-week intervention period, while in the control arm schools kept their regular canteen menu.

### 2.1. Participants

Schools were eligible if they met the following inclusion criteria:

- Government, independent and Catholic primary schools (Kindergarten–Year 6) with a school canteen;
- School is located in the Greater Sydney Area;
- School canteen is using the online ordering system of a specific online provider;
- School canteen is operating at least 3 days a week;
- Principals are willing to let their school participate and are willing to share de-identified sales data (provided by the online ordering provider);
- Canteen managers are willing to implement changes to their vegetable provision and participate in a follow-up survey.

Exclusion criteria were:

- Schools registered as a Special Needs school;
- Combined schools (K-12).

A sample size of 16 schools was sought from a range of socio-economic status (SES) levels. The sample size was calculated for schools (not students) by using G*Power (version 3.1.9.4) and was based on a repeated measures analysis with two groups, 9 time points (baseline and 8 intervention weeks), a desired effect size of F = 0.4 (being between a medium (0.3) and a large (0.5) effect size, G*Power version 3.1.9.4), α = 0.05, power = 0.80 and non-sphericity correction of 1 (meaning the assumption of sphericity was not violated) [30]. For feasibility reasons and to reduce differences between schools, larger canteen operators servicing multiple primary schools were targeted.

Informed consent was obtained from school principals and canteen managers. All study procedures were approved by the CSIRO Human Research Ethics Committee (2020_120_LR), the NSW Department of Education (government schools) and the relevant Catholic Education Dioceses (Catholic schools). This study was prospectively registered with the Australian New Zealand Clinical Trials Registry (ACTRN12621000564853).

### 2.2. Multi-Strategy Behavioral Intervention

The intervention consisted of an 8-week multi-strategy behavioral intervention to increase vegetable offerings and their salience in primary school canteens. The strategies were co-designed with Healthy Kids Association, a not-for-profit organization offering a wide range of services targeting school canteens, and two canteen operators each being responsible for menu design of multiple schools. The following strategies were deployed:

1. Increased presence of vegetable offerings:
  - A novel vegetable-containing product, ‘Rainbow Dippers’, was introduced. The product was designed and produced specifically for this study. It consisted of a vegetable-based dip and vegetable dipper (raw vegetable sticks) and was designed to contain a minimum of one serve (75 g) of vegetables. Four different flavor/color combinations were developed (e.g., carrot and celery sticks with a beetroot dip, cucumber and capsicum sticks with an avocado dip) with each combination for sale for 2 weeks during the 8-week intervention period. This product concept was positively evaluated in a qualitative study with children [20] and aligned with the CAMPOV model [20] by using bright colors, a variety of colors, bite-sized pieces, providing a fun eating experience through the dipping and using familiar and well-accepted snack vegetables. The components of the product were based on sensory consumer acceptance testing with a separate group of school aged children, with all variants being well accepted by children (data not shown). The product adhered to schools’ guidelines concerning allergies and was produced in a commercial production facility specifically for this study.
  - Increased vegetable content in existing popular hot meals and foods on the canteen menu, aligning with the CAMPOV model [20] by pairing vegetables with liked and familiar foods and flavors. Canteen managers were provided with advice and support on how the vegetable content of their most popular hot meals could be increased.
2. Changes to the online menu structure/choice architecture:
  - Positioning changes were made on online menus to fit with the “primacy effect” [26,28]. Vegetable-containing items were positioned first on the online menu, ahead of non-vegetable containing items listed in the same category.
  - Changes to the default: A recent menu analysis showed that 60% of sandwiches did not contain vegetables, with many having an option to additionally add (‘opt-in’) salad vegetables [11]. Based on the prospect theory, the strategy of having a salad vegetable on sandwiches as a default setting was developed. All protein-based (e.g., cheese/ham/turkey/chicken) sandwiches were served with a salad vegetable by default on the online menu, with the option for parents/students to opt-out.
3. A brochure with additional suggestions to increase vegetable offerings. Canteen managers were provided with a 12-page brochure containing information and suggestions to increase the vegetable content of their menu. Suggestions were based on the results of the menu analysis of vegetable offerings on canteen menus [11] and included, among others, “Adding veggies to dishes children already love” (e.g., pizza, hot meal, sandwiches and salads), new vegetable items (snacks, meal deals) and promotion suggestions (focus on fun and taste testing with students to select better recipes). This brochure aligned with the CAMPOV model [20] by focusing on the importance of sensory acceptance and fun, and not focusing on the health benefits of increased vegetable consumption.

Canteen managers allocated to the intervention arm of the study were provided with an overview of the strategies and brochure (communicated as the VegUP strategies) which were discussed with a member of the research team. Canteen managers could decide which additional strategies from the brochure would suit their canteens and if certain strategies would not be suitable. If canteen managers felt they needed to withdraw a food item to comply with their menu policy (e.g., if the addition of a new snack would require the removal of another snack) they were encouraged to implement this, and the change was recorded. Intervention adherence was checked by recording which initial strategies were implemented, and by a phone call mid-way through and at the end of the intervention to determine whether these strategies were maintained. Fidelity checks further included weekly checks of the online menu (both intervention and control schools) and delivery records of Rainbow Dippers supplied by the commercial provider. Canteen managers were also asked to send pictures of intervention items in the first intervention week.

Parents and students were informed about the menu changes at the beginning of the school term in which the intervention was implemented via the school’s regular communication channels (e.g., newsletter, social media, or online ordering portal) through a “Term Specials” A4 leaflet summarizing the changes. Further, Rainbow Dippers were advertised on a top banner of the online ordering portal as “Rainbow Dippers—A colorful tasty snack of vegetable sticks and dip, with a new combination every 2 weeks! See the Snacks section.” The communication to parents emphasized sensory claims and fun, and de-emphasized health messages, in line with the CAMPOV model [20].

### 2.3. Procedure

The provider of an online school canteen ordering system provided the researchers with a list of schools and canteen operators in the Greater Sydney area they serviced. Canteen operators were ranked according to the number of primary schools they serviced and approached sequentially. When the canteen operator expressed interest, invitation emails were sent to the school principal and canteen manager of the schools they serviced. Once schools were recruited into the study, they were randomly allocated to one of the two treatments (intervention or control). A randomized block design, with SES levels as blocks, was created for the purpose of generating the allocation sequence by an independent statistician prior to recruitment. Allocation to treatment was carried out by the principal investigator in the order of enrolment date, thus allocation concealment was conducted using sequential dates. Canteen managers of intervention schools were instructed not to change their menu or current practices during the baseline period, to implement changes during the intervention period and not to discuss any intervention strategies with canteen managers allocated to the control arm of the study. The changes to the online menu were made by the online ordering provider. Rainbow Dippers were supplied free of charge to the intervention schools. Canteen managers of control schools were instructed not to change anything in their menu during both the baseline and follow-up period and to maintain the status quo about other practices.

Baseline data were collected in school term 2 (April–July) of 2021. The intervention was scheduled to take place the following school term but was delayed due to COVID-related school closures and measures. Hence, intervention implementation was conducted in school term 1 (February–April) of the following year (2022).

### 2.4. Measures

The primary outcome was the difference in vegetables sold (g/week) from baseline to follow-up period in the intervention versus control canteens. Baseline data were collected during a 3-week period and follow-up data were collected for 8 weeks, which coincided with the weeks the intervention was implemented in the intervention group. Canteen sales data were used in a sequential process:

- Menus of participating schools were assessed by a trained dietitian (S.D.) with over 5 years of experience in working with school canteens and reviewing school canteen menus according to the NSW Health and Department of Education guidelines [9]. Vegetable content (in g) was determined for each item on the online menu using information about ingredients, recipes and brands from the canteen manager.
- De-identified online sales data were provided by the online provider. Lunch orders in the categories ‘special events’ and ‘birthday buckets’ were excluded from the analysis as they related to group orders and not individual student orders. Brown-paper-bag orders (i.e., manually written orders typically submitted in a brown paper bag over-the-counter, rather than online orders) and over-the counter-sales (i.e., students buying foods at the counter at recess or after lunch) of any vegetable-containing items were recorded daily on a paper checklist by canteen managers.
- Vegetables sold (in grams) were calculated by multiplying the number of vegetable-containing items sold each day in the canteen by the vegetable content (in grams) of those items. Daily vegetable sales were converted to weekly sales by summation of daily totals.

Secondary outcomes were:

- Vegetable sales data in subcategories of a typical school canteen menu structure [11] were analyzed using the procedures described above to determine whether the intervention affected specific subcategories of foods more than others.
- Canteen revenue: to determine that the intervention did not adversely (negatively) affect canteen revenue, total online canteen revenue data (in Australian dollars) were analyzed.
- Vegetable waste: this study assumed that vegetable sales are a good approximation of vegetable intake. To verify this assumption, student vegetable waste was collected from school grounds from a random sample of 4 participating schools (2 intervention and 2 control schools; one medium/high and one low SES school in each arm). Thus, this study considers plate waste and does not consider non-served foods as a source of waste [31]. Vegetable waste was measured over two representative days mid-baseline and the mid-intervention period. Dedicated bins and waste bags were provided during test days. Waste was collected by canteen managers and handed to research staff. On the same day, waste was sorted for vegetable items and weighed, using a systematic methodology based on procedures described by Boschini et al. [31]. Total waste (food and packaging) (A&D Personal Precision Scale, UC-321) and vegetable waste (Mettler Toledo Precision Balance, ME4002) were weighed and recorded.
- Acceptability of the intervention strategies and implementation of the multi-strategy intervention itself was evaluated through an online quantitative survey that intervention canteen managers completed after the intervention (Supplementary Materials). They responded to their level of agreement using 5-point Likert scales on five aspects of each of the intervention strategies: ease of implementation, if children responded positively to the strategy, if it was time-consuming or labor-intensive, if it was wasteful, and sustainability for the future. They also rated level of agreement to perceived factors influencing implementation based on the validated “Theoretical Domains Framework Questionnaire for Implementation (TDF)” [32] and the “Measurement Instrument for Determinants of Innovations (MIDI)” [33]. This included a total of 27 perceived individual (determinants including knowledge, self-efficacy, motivation and attitude) and environmental (determinants including the need for support, innovation and organizational support) factors (modified from [34]) (Supplementary Table S1). This was followed up with a short interview to gather qualitative information about their responses.

Background data collected included total student enrolments and sector (government or non-government) obtained from a national school website [35], SES level of a school’s suburb derived from the Index of Relative Socio-Economic Advantage and Disadvantage scores (IRSEAD) [36], number of days the canteen was operating and other nutrition related education programs running at the school gathered from school staff.

### 2.5. Statistical Analysis

Statistical analyses were conducted using SPSS (Version 28.0.1.0, IBM Corp, Armonk, NY, USA). A correction factor was applied to sales data to account for year-to-year changes in student enrolment numbers using student enrolment data from 2020 [35] and 2021 [37] (e.g., if student enrolments had increased by 5% a division factor of 1.05 was applied to follow-up data).

For the days that canteens were not open (e.g., due to public holidays or pupil-free days) the average vegetable sales for that day was imputed by calculating the average sales on that weekday from other weekdays in that period (baseline or follow-up) from the same school. This was performed because all analyses were based on weekly sales (Monday through to Friday).

Vegetable sales data (g/week) and total sales (AUD/week) were analyzed using linear mixed-model analysis with treatment arm (intervention or control), time point (baseline or follow-up period) and their interaction as fixed factors, and with adjustments for SES level (low/medium/high) and sector (government or non-government) as fixed factors, and for student enrolment number at baseline as covariate. No random factors or intercept were included in the model. This analysis was a deviation from the original protocol, which described a repeated-measures analysis with baseline data averaged across three weeks. However, as there was no reason to assume a specific trend (increase or decrease) across the intervention period, the current analysis was considered to better compare differences between baseline and follow-up, taking all 11 points in time into account.

Vegetable waste data (g) was analyzed using linear mixed-model analysis with treatment arm (intervention or control) and time point (baseline or follow-up period) and their interaction as fixed factors, and the same adjustments as used in the other models, to test if a change in vegetable waste was not significantly greater in the intervention group compared to the control group.

## 3. Results

### 3.1. Participants

The first canteen operator approached was interested to participate and fulfilled the eligibility criteria, therefore schools they serviced were approached. A total of eight intervention and eight control schools from this canteen operator were included in the study (Table 1). The intervention group contained three low-, three medium-, and two high-SES schools, while the control school contained only two low-SES schools and included three medium and three high-SES schools. There was also a small difference in school sector, with the intervention group containing five government and three non-government schools and the control group containing six government and two non-government schools. Eighty-seven percent of schools took part in the Crunch & Sip program (a fruit and vegetable break) and four schools had a school garden, with representation equal across intervention and control schools. All canteens were operating five days a week. Schools and their canteens were closed for a small number of days during the study period due to e.g., a public holiday or pupil-free day. A total of 4302 students placed at least one order in the baseline period, and 5577 students in the follow-up period, with a weekly average of 396 ± 191 lunch orders in intervention schools, and 505 ± 337 lunch orders in control schools, at baseline. The menus at baseline were almost identical, with only minor differences between schools. Menus consisted of a typical Australian canteen menu [11] with the categories of burgers (e.g., beef burger, chicken burger), pasta/rice dishes (e.g., spaghetti Bolognese, butter chicken), sandwiches, rolls and wraps (e.g., cheese sandwich), hot foods (i.e., hot foods other than pasta/rice dishes and burgers, e.g., chicken nuggets, sausage roll), sushi, salads (e.g., garden salad), drinks (e.g., milk, juice), cold treats (e.g., frozen ice blocks) and snacks. The snacks subcategory consisted of a combination of sweet (e.g., fruit, fruit salad, banana bread) and savory (e.g., garlic bread). An average of 78 ± 1 food and drink items were offered, with an average of 8.7 ± 4.4 items per subcategory.

### 3.2. Intervention Implementation

All intervention canteens implemented the strategies of making online positioning changes to vegetable-containing items, introducing the Rainbow Dippers (vegetable content from four variants ranging from 78 to 109 g), increasing the vegetable content of popular hot meals and adding a default vegetable to sandwiches (with option to opt-out). Spaghetti Bolognese (the addition of grated carrot and zucchini increased the vegetable content from 43 to 58 g per serve) and butter chicken (the addition of diced sweet potato increased the vegetable content from 0 to 25 g per serve) were the hot meals in which the vegetable content was increased. Three canteens included lettuce (50 g) as the default vegetable in all protein-based sandwiches, wraps and rolls. Five canteens included lettuce (50 g, 3 days/wk) or cucumber (60 g, 2 days/wk) in all protein-based sandwiches, wraps and rolls on an alternating schedule. Based on ideas in the brochure, the canteen operator suggested additional strategies which were discussed with intervention canteen managers. All intervention canteens also added beetroot (40 g) as default to all burgers (with the option to opt-out), added a slice of tomato (8 g) to a ‘pizza muffin’ (a savory muffin with ham and cheese; for some schools this was a new item) and fruit was replaced by cherry tomatoes and carrot sticks (total 45 g of vegetables) in a Bento Box (a variety box including a sandwich, chicken tender and snacks). Five (medium-/high-SES) schools sold the Rainbow Dippers for AUD 2.50, whereas three (low-SES) schools sold the Rainbow Dippers for AUD 2. No items were removed from the menu because of the introduction of intervention items, and the prices of menu items were not altered.

### 3.3. Intervention Fidelity

The mid-term and end-of-intervention fidelity phone calls indicated that all strategies were maintained throughout the 8-week intervention period. The weekly online menu fidelity checks showed that positioning changes, the addition of default vegetables to burgers and sandwiches, the altered Bento Boxes, two-weekly rotation of Rainbow Dippers and advertisements were all consistently applied in the intervention schools. Rainbow Dipper delivery dockets confirmed correct flavor rotations were delivered to the schools as per the delivery schedule. Photographs of intervention dishes were provided by 38% (3/8) of canteen managers. The online menu fidelity checks in control schools showed that menus were not changed.

### 3.4. Primary Outcome Measure: Vegetable Sales

**Figure 1:**
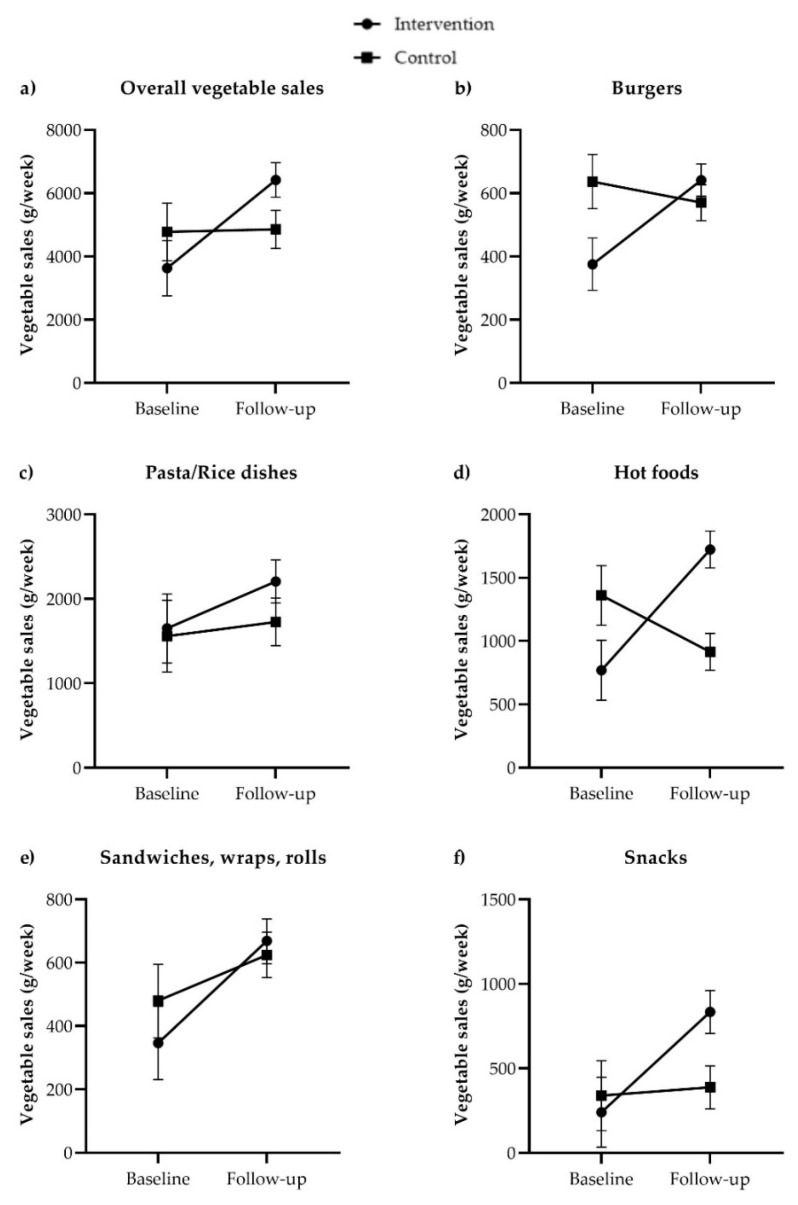
Estimated mean values with standard error of the mean (SEM) on baseline and follow-up in the intervention group (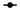) and in the control group (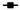) for overall vegetable sales (**a**), and vegetable sales per menu subcategory (**b**–**f**) in grams of vegetables/week based on the linear mixed models.

The estimated overall vegetable sales in grams per week based on the linear mixed-model analysis are shown for the baseline and follow-up period for both the intervention and control group (Table 2) and visually represented in Figure 1. The estimated average vegetable sales for the intervention group increased from 3630 to 6418 g/week, whereas the estimated average sales for the control group increased from 4775 to 5167 g/week. The effect in the intervention group was significantly (*p* < 0.001) larger than in the control group, with a differential effect (i.e., the difference between follow-up and baseline in the intervention group, adjusted for differences in the control group during that time period) of +2707 g/week (CI 1276–4137) in overall vegetable sales. This equated to an increase of 75% in vegetable sales in intervention schools compared to baseline, when adjusted for differences in control schools during that time period.

### 3.5. Secondary Outcome Measures

#### 3.5.1. Vegetable Sales in Subcategories

The difference in vegetable sales was significantly different between intervention schools as compared to the control schools from baseline to follow-up period in three of the five subcategories where intervention strategies were included: burgers (*p* < 0.001), hot foods (*p* < 0.001) and snacks (*p* = 0.002) (Table 2). In the burgers subcategory, estimated vegetable sales increased in intervention schools by 332 g/week (CI 198–467) more than control schools, which was an increase of 89% in intervention schools compared to baseline, when adjusted for differences in control schools during that time period. The category of hot foods (where fruit was replaced by vegetables in Bento Boxes) increased by 1399 g vegetables per week (CI 1008–1790), an increase of 182%. The category snacks (where Rainbow Dippers were introduced and pizza muffins included tomato) increased by 544 g vegetables per week (CI 201–886), an increase of 226% from baseline. In the other two categories, pasta/rice dishes and sandwiches, the treatment by time effect was not significant (*p* = 0.25 and *p* = 0.07, respectively), indicating there were no differences in changes in sales between intervention and control schools. However, a significant time effect was seen, indicating that compared to the baseline period, sales in those categories increased in both intervention and control schools (*p* = 0.03 and *p* < 0.001, respectively). In the category pasta/rice dishes (where the vegetable content was increased in butter chicken and pasta Bolognese in the intervention schools), the sales increased by 34% in intervention schools and by 11% in control schools. Further, in the sandwiches, wraps and rolls, where a differential effect of 176 g/week (CI −16–367) was observed in intervention schools which just failed to reach significance (*p* = 0.07), vegetable sales increased by 93% in intervention schools and by 30% in control schools.

#### 3.5.2. Sales Revenue

The effect on total sales revenue was measured as a potential adverse effect. There was a significant treatment by time effect in favor of the intervention schools (a differential effect of AUD 387/week (CI 70–705). Table 2 shows that revenue remained stable at intervention schools and decreased at control schools. Therefore, no adverse effect of the vegetable intervention strategies on the weekly online sales revenues in dollars was seen.

#### 3.5.3. Vegetable Waste

Table 3 shows that total waste (food and packaging) measured at the school level ranged from an estimated average of 2.4 to 7.1 kg per day and there was no change in total waste as a result of the intervention (*p* = 0.38). Vegetable waste represented only 0–3% of total waste at all time points. A differential effect of 2 g/week (CI 20–220) in the intervention compared to control schools was observed (*p* = 0.98), indicating there was no change in the amount of vegetable waste as a result of the intervention.

#### 3.5.4. Acceptability of the Intervention

Canteen managers found the multi-strategy behavioral intervention easy to implement with no major barriers at the individual and environmental level (Supplementary Materials). The specific intervention strategies were perceived as easy to implement and not time consuming. Strategies were also perceived as not wasteful with the exception of the novel Rainbow Dippers. Canteen managers felt that children were positive about the strategies of increasing the vegetable content in the pasta/rice dishes, Bento Boxes and pizza muffins, neutral about vegetables added to sandwiches and online ordering changes, and felt children did not respond positively to the Rainbow Dippers and beetroot in burgers. Beetroot is a ‘classic’ vegetable on burgers in Australia, however, not commonly added to burgers in fast-food chains. Qualitative information from the canteen managers suggested this appeared to be the frame of reference for this younger population. Canteen managers felt that Rainbow Dippers were not a sustainable strategy for the future with other strategies scoring around the neutral point (Supplementary Materials). They suggested the use of a familiar and highly liked (non-vegetable based) dip for the Rainbow Dippers may be considered to increase appeal amongst children.

## 4. Discussion

This study investigated the effect of a multi-strategy behavioral intervention to increase vegetable offerings and their salience on the vegetable sales in primary school canteens. Vegetable sales significantly increased in school canteens where the intervention strategies were implemented, in comparison with control canteens that continued to implement their regular menu. Significant increases as a result of the intervention were observed in the subcategories of burgers, other hot foods and snacks, but not in the subcategories of sandwiches and pasta/rice dishes. There was no adverse effect on vegetable waste or sales revenue. Canteen managers felt that implementation of the intervention was easy, with children responding favorably to three of the strategies and two being perceived as neutral.

Previous systematic reviews on school-based interventions aiming to increase fruit and vegetable intake including classroom based curriculum lessons, free distribution of fruit and vegetables, provisioning, and/or school garden programs showed modest effect sizes [16,38]. However, when separating meta-analysis outcomes for fruit and vegetable intake, school-based interventions were found to merely increase fruit intake, with only 0.07 serves increase in vegetable intake observed [16]. None of these studies used sales as an outcome measure, therefore the results cannot be directly compared There are few canteen interventions with which the current study can be directly compared. Wyse et al. [18] similarly found that a multi-strategy behavioral school canteen intervention was successful in changing purchase behavior amongst students. This study used multiple online strategies to increase the salience of healthy foods, including menu labeling, positioning, prompting and feedback, and showed these changes were effective in improving the nutritional quality of lunch orders in terms of overall energy content and saturated fat content. A study that specifically investigated positioning fruit and vegetable snacks as the first and last items in an online menu found no effect on the selection of those snacks [19], but in the current study, the effect of this positioning strategy could not be separated from the other strategies.

This study was a multi-strategy behavioral intervention, therefore the contribution of individual components of the strategy cannot be individually assessed, as in most menu subcategories, both the strategy of online positioning as well as changes in the vegetable offering were implemented. Nonetheless, subcategory sales provide additional insights. Increases in vegetable sales compared to baseline were observed in all subcategories, ranging from 34 to 246%, although they were only statistically higher compared with control schools in the categories of snacks (where strategies of tomato added to the pizza muffin and Rainbow Dippers were introduced), other hot foods (vegetables in Bento Boxes) and burgers (beetroot added to burgers). These results make the incremental impact across a number of product categories clear, and confirm the need for a multiple-component, multiple-eating-occasion, multiple-product approach, which is in line with best practice guidelines for increasing children’s vegetable intake [39].

Vegetable sales increased over time in both treatment arms in the categories of sandwiches and pasta/rice dishes. The reason remains unclear, but it may be related to seasonal changes. Although there was no significant interaction between treatment arm and time, in those categories, the percentage increase from baseline was numerically larger in intervention than control schools. Schools typically have more vegetable-containing items in these categories than in some others, like snacks [11], and therefore relative increases may have been more incremental. We also cannot offer a suitable explanation why sales revenue remained stable in the intervention schools but not in control schools.

There seemed to be differences between intervention and control schools in several outcome measures at baseline, despite menus at baseline being similar. Vegetable sales appeared numerically lower at baseline in intervention than control schools. Arguably, this offered more potential for change in intervention schools, but at the same time it could be argued that there were larger barriers at these schools in place (e.g., children’s preferences) which are more difficult to overcome. Although schools were randomly allocated to a group, significantly contributing factors (SES level, the school sector and student enrolment numbers at baseline) were included in the linear mixed models to control for these factors.

Canteen managers felt that no major barriers at the individual or environmental level were encountered to implement the multi-strategy intervention. The specific intervention strategies were perceived as easy to implement, not time-intensive or wasteful (with the exception of Rainbow Dippers), indicating that these operational barriers to increasing children’s vegetable consumption in canteens can be mostly overcome. Wastefulness of Rainbow Dippers related to the difference between product delivered and sold, and therefore an item like this may be better prepared by canteen managers on-site so that volumes can be better tailored to demand. The canteen managers felt that children responded positively to vegetables in pasta/rice dishes, Bento Boxes and pizza muffins, and were neutral towards online positioning changes and vegetables in sandwiches. Therefore, the barrier of ‘children’s preferences’ can be partially overcome. This was also supported by the vegetable waste data, as the intervention strategies did not lead to increased vegetable waste. Canteen managers reported that children did not respond positively to beetroot in burgers or the Rainbow Dippers. Changes to these strategies may make their evaluation amongst children more favorable. For example, another type of vegetable could be considered as the default addition on burgers. Rainbow Dippers, being a snack product, compete with highly palatable snacks, with energy-dense, nutrient-poor foods being the most commonly purchased foods in Australian school canteens [40,41]. Therefore, a snack product containing one serve of vegetables may not be sustainable/commercially viable in a school canteen environment.

Canteen managers felt that adding beetroot to burgers was less successful amongst children, however, increases in vegetable sales were observed in the burger category. A discrepancy between actual and perceived effect was observed in the multi-strategy behavior intervention study by Wyse et al. [18], which showed an improved nutritional quality but only 43% of canteen managers thought the intervention had an effect on online purchases. These observations indicate that it is important to educate canteen managers on the evidence base when interventions are being rolled out on a larger scale, as any positive effects may not be readily visible to canteen managers at the individual school level, which may impact their motivation to sustain interventions.

This study found no increase in student vegetable waste as a result of the intervention. This measure of waste included student waste both from lunch orders and food brought from home, and therefore vegetable waste from the lunch orders and vegetable waste from food brought from home could not be differentiated. The researchers considered implementing this by setting up different bins, but it was decided it would threaten ecological validity and would be too onerous on school staff to separate these waste streams. Therefore, it is not possible to directly calculate vegetable consumption from the vegetable sales and vegetable waste data. However, these results suggest that vegetable sales can be a good proxy indicator of vegetable consumption as the students did not discard more vegetables than before.

A strength of the current study was its robust study design, the inclusion of both online and over-the-counter sales, the collection of vegetable waste data demonstrating that sales can be used as a proxy for consumption, the spread in socio-economic status of schools and the inclusion of both government and non-government schools. A limitation of this study is the external representativeness, as all schools were in the Greater Sydney area and operated by one commercial canteen operator, which meant that canteen menus were very similar. However, they were representative of typical Australian canteen menus in terms of menu structure and number and type of foods offered [11]. The use of a single provider also provides a potential contamination risk. However, this study did not involve training of canteen managers with knowledge that accidentally could be disclosed, rather provision of offerings and menu architecture changes which could clearly be kept separately to the intended schools. Furthermore, a simple imputation method was used to adjust missing vegetable sales data due to school closures, and more elaborate methods (e.g., multiple imputation by chained equations) could be considered.

Further research reflecting the diversity of Australian school eating environments, including primary school canteens with more varied menus, different operating systems (e.g., run by schools themselves or by Parent & Citizen Committees) and in other states is recommended to determine whether a similar effect on vegetable sales is observed. Economic feasibility is also a key factor that needs to be considered when considering the scalability of the intervention. Prices of foods were kept the same in the current study. Where an increase in vegetable content can be accompanied by a decrease in other ingredients such as meat (such as in pasta and rice dishes), cost neutrality may be achieved. However, when vegetables are added (e.g., adding beetroot to burgers) without further ingredient changes, this will decrease the profitability of the item, and pricing changes to the item or a relative pricing strategy to the menu may need to be considered to compensate for increased costs. Further research could also investigate the effect of increasing vegetable sales on the sales of other food groups (e.g., dairy or fruit) or foods with specific sensory properties (e.g., sweet snacks) to understand the effect of vegetable-focused interventions on other aspects of dietary quality.

In conclusion, the current study showed that a multi-strategy behavioral intervention involving vegetable offerings and menu architecture can positively influence vegetable sales, without negatively impacting vegetable waste or sales canteen revenue in primary school canteens.

## Figures and Tables

**Table 1:** Characteristics of participating schools who either implemented the multi-strategy behavioral intervention canteen strategies (intervention schools) or continued to offer their regular canteen menu (control schools).

Characteristics	Intervention Schools (*n* = 8)	Control Schools (*n* = 8)
Socio-economic status (*n*) ^1^		
Low	3	2
Medium	3	3
High	2	3
School sector (*n*)		
Government	5	6
Non-government	3	2
Students enrolled at school at baseline (*n*) ^2^	504 ± 259	562 ± 503
Canteen regular operating days/week	5 ± 0	5 ± 0
Number of weekly lunch orders (at baseline)	396 ± 191	505 ± 337
Days/week school closed during baseline period	0.5 ± 0.2	0.4 ± 0.2
Days/week school closed during follow-up period	0.1 ± 0.2	0.1 ± 0.1

^1^ Based on Index of Relative Socio-Economic Advantage and Disadvantage (IRSEAD) scores from the Australian Bureau of Statistics [36], Low = IRSEAD deciles 1–4, medium = IRSEAD deciles 5–8, high = IRSEAD deciles 9–10. ^2^ MySchool data [35].

**Table 2:** Primary and secondary outcomes in the baseline and follow-up period for schools that either implemented the multi-strategy behavioral intervention (intervention schools) or continued to offer their regular canteen menu (control schools).

Variable	Baseline, Mean (95% CI)		Follow-Up, Mean (95% CI)		Intervention Versus Control	
	Intervention (*n* = 8 Schools)	Control (*n* = 8 Schools)	Intervention (*n* = 8 Schools)	Control (*n* = 8 Schools)	Group-by-Time Differential Effect (95% CI) ^1^	*p* Value ^1^
**Primary outcome**						
Overall vegetable sales (in g/week)	3630 (2757, 4503)	4775 (3865, 5685)	6418 (5872, 6964)	4856 (4253, 5458)	2707 (1276, 4137)	<0.001
**Secondary outcomes**						
*Vegetable sales (in g/week) in subcategories*						
Burgers	375 (293, 458)	636 (551, 722)	641 (590, 692)	570 (513, 626)	332 (198, 467)	<0.001
Pasta/rice dishes	1650 (1242, 2058)	1558 (1133, 1983)	2206 (1951, 2461)	1726 (1445, 2008)	388 (−280, 1056)	0.25
Hot foods	769 (532, 1005)	1360 (1124, 1596)	1722 (1577, 1869)	914 (769, 1059)	1399 (1008, 1790)	<0.001
Sandwiches, wraps, rolls	346 (231, 462)	480 (363, 595)	669 (596, 738)	624 (553, 696)	176 (−16, 367)	0.07
Snacks	241 (34, 448)	339 (132, 546)	834 (707, 960)	388 (261, 514)	544 (201, 886)	0.002
*Sales revenue (in AUD/week)*	1546 (1352, 1739)	2016 (1814, 2219)	1701 (1579, 1822)	1783 (1649, 1916)	387 (70, 705)	0.02

^1^ Linear mixed-model analysis using change between baseline and follow-up, adjusted for SES level, the school sector and student enrolment numbers at baseline. The differential effect is the difference between groups, adjusted for baseline.

**Table 3:** Secondary outcome of student vegetable waste in the baseline and follow-up period for schools who either implemented the multi-strategy behavioral intervention (intervention schools) or continued to offer their regular canteen menu (control schools).

Variable	Baseline, Mean (95% CI)		Follow-Up, Mean (95% CI)		Intervention Versus Control	
	Intervention (*n* = 2 Schools)	Control (*n* = 2 Schools)	Intervention (*n* = 2 Schools)	Control (*n* = 2 Schools)	Group-by-Time Differential Effect (95% CI) ^1^	*p* Value ^1^
Total waste (kg/day)	5.4 (0.9, 9.9)	3.6 (−0.8, 8.1)	7.1 (2.6, 11.6)	2.4 (−2.1, 6.9)	3.0 (−4.2, 10.2)	0.38
Vegetable waste (g/day)	107 (−29, 243)	−23 (−160, 112)	182 (46, 318)	49 (−87, 185)	2 (−216, 220)	0.98

^1^ Linear mixed-model analysis using change between baseline and follow-up, adjusted for SES level, the school sector and student enrolment numbers at baseline. The differential effect is the difference between groups, adjusted for baseline.

## Data Availability

Not applicable.

## Supplementary Materials

The following supporting information can be downloaded at: https://www.mdpi.com/article/10.3390/nu14194218/s1, Table S1: Questionnaire statements that canteen managers responded to measuring their level of agreement with individual and environmental factors affecting multi-strategy intervention implementation. Table S2: Individual and environmental factors affecting multi-strategy intervention implementation as perceived by the canteen managers, shown as means and standard deviations (SD). Table S3: Canteen manager evaluation of seven specific intervention strategies on five acceptability factors, shown as means and standard deviations (SD).

## References

[1] Aune D., Giovannucci E., Boffetta P., Fadnes L.T., Keum N., Norat T., Greenwood D.C., Riboli E., Vatten L.J., Tonstad S. (2017). Fruit and vegetable intake and the risk of cardiovascular disease, total cancer and all-cause mortality—A systematic review and dose-response meta-analysis of prospective studies. Int. J. Epidemiol..

[2] Boeing H., Bechthold A., Bub A., Ellinger S., Haller D., Kroke A., Leschik-Bonnet E., Müller M.J., Oberritter H., Schulze M. (2012). Critical review: Vegetables and fruit in the prevention of chronic diseases. Eur. J. Nutr..

[3] World Health Organisation Increasing Fruit and Vegetable Consumption to Reduce the Risk of Noncommunicable Diseases.

[4] Kim S.A., Moore L.V., Galuska D., Wright A.P., Harris D., Grummer-Strawn L.M., Merlo C.L., Nihiser A.J., Rhodes D.G. (2014). Vital signs: Fruit and vegetable intake among children—United States, 2003–2010. Morb. Mortal. Wkly. Rep..

[5] Lynch C., Kristjansdottir A.G., Te Velde S.J., Lien N., Roos E., Thorsdottir I., Krawinkel M., de Almeida M.D.V., Papadaki A., Ribic C.H. (2014). Fruit and vegetable consumption in a sample of 11-year-old children in ten European countries–the PRO GREENS cross-sectional survey. Public Health Nutr..

[6] Australian Institute of Health and Welfare (2018). Nutrition Across the Life Stages.

[7] Manson A.C., Johnson B.J., Zarnowiecki D., Sutherland R., Golley R.K. (2021). The food and nutrient intake of 5-to 12-year-old Australian children during school hours: A secondary analysis of the 2011–2012 National Nutrition and Physical Activity Survey. Public Health Nutr..

[8] Lucas P.J., Patterson E., Sacks G., Billich N., Evans C.E.L. (2017). Preschool and School Meal Policies: An Overview of What We Know about Regulation, Implementation, and Impact on Diet in the UK, Sweden, and Australia. Nutrients.

[9] NSW Ministry of Health (2017). The NSW Healthy School Canteen Strategy—Food and Drink Benchmark.

[10] Queensland Government (2020). Smart Choices Healthy Food and Drink Supply Strategy for Queensland Schools.

[11] Beelen J., Heffernan J.E., Cochet-Broch M., Djakovic S., Chung D., Golley R.K., Poelman A.A. (2021). Menu audit of vegetable-containing food offering in primary school canteens in Sydney, Australia: A preliminary study. Int. J. Environ. Res. Public Health.

[12] Bell A., Swinburn B. (2004). What are the key food groups to target for preventing obesity and improving nutrition in schools?. Eur. J. Clin. Nutr..

[13] Chawner L.R., Blundell-Birtill P., Hetherington M.M. (2021). Predictors of vegetable consumption in children and adolescents: Analyses of the UK National Diet and Nutrition Survey (2008–2017). Br. J. Nutr..

[14] Cullen K.W., Chen T.-A. (2017). The contribution of the USDA school breakfast and lunch program meals to student daily dietary intake. Prev. Med. Rep..

[15] Johnston C.A., Moreno J.P., El-Mubasher A., Woehler D. (2012). School lunches and lunches brought from home: A comparative analysis. Child. Obes..

[16] Evans C.E., Christian M.S., Cleghorn C.L., Greenwood D.C., Cade J.E. (2012). Systematic review and meta-analysis of school-based interventions to improve daily fruit and vegetable intake in children aged 5 to 12 y. Am. J. Clin. Nutr..

[17] Van Cauwenberghe E., Maes L., Spittaels H., Van Lenthe F.J., Brug J., Oppert J.-M., De Bourdeaudhuij I. (2010). Effectiveness of school-based interventions in Europe to promote healthy nutrition in children and adolescents: Systematic review of published and ‘grey’literature. Br. J. Nutr..

[18] Wyse R., Delaney T., Stacey F., Zoetemeyer R., Lecathelinais C., Lamont H., Ball K., Campbell K., Rissel C., Attia J. (2021). Effectiveness of a multistrategy behavioral intervention to increase the nutritional quality of primary school students’ web-based canteen lunch orders (click & crunch): Cluster randomized controlled trial. J. Med. Internet Res..

[19] Wyse R., Gabrielyan G., Wolfenden L., Yoong S., Swigert J., Delaney T., Lecathelinais C., Ooi J.Y., Pinfold J., Just D. (2019). Can changing the position of online menu items increase selection of fruit and vegetable snacks? A cluster randomized trial within an online canteen ordering system in Australian primary schools. Am. J. Clin. Nutr..

[20] Poelman A.A., Heffernan J.E., Cochet-Broch M., Beelen J. (2021). Development and Proof-of-Concept Evaluation of a Sensory Science-Based Model for Product Development of Vegetable-Based Products for Children. Foods.

[21] Poelman A.A., Delahunty C.M., de Graaf C. (2017). Vegetables and other core food groups: A comparison of key flavour and texture properties. Food Qual. Prefer..

[22] Hetherington M.M., Chawner L.R. (2022). From food preference development to responsive feeding–Selective studies to commemorate the life and work of Dr Leann Birch. Appetite.

[23] Bell L.K., Gardner C., Tian E.J., Cochet-Broch M.O., Poelman A.A., Cox D.N., Nicklaus S., Matvienko-Sikar K., Daniels L.A., Kumar S. (2021). Supporting strategies for enhancing vegetable liking in the early years of life: An umbrella review of systematic reviews. Am. J. Clin. Nutr..

[24] Hollands G.J., Shemilt I., Marteau T.M., Jebb S.A., Kelly M.P., Nakamura R., Suhrcke M., Ogilvie D. (2013). Altering micro-environments to change population health behaviour: Towards an evidence base for choice architecture interventions. BMC Public Health.

[25] Skov L.R., Lourenco S., Hansen G.L., Mikkelsen B.E., Schofield C. (2013). Choice architecture as a means to change eating behaviour in self-service settings: A systematic review. Obes. Rev..

[26] Bucher T., Collins C., Rollo M.E., McCaffrey T.A., De Vlieger N., Van der Bend D., Truby H., Perez-Cueto F.J. (2016). Nudging consumers towards healthier choices: A systematic review of positional influences on food choice. Br. J. Nutr..

[27] Thaler R.H., Sunstein C.R., Balz J.P. (2013). Choice Architecture.

[28] Mantonakis A., Rodero P., Lesschaeve I., Hastie R. (2009). Order in choice: Effects of serial position on preferences. Psychol. Sci..

[29] Tversky A., Kahneman D. (1991). Loss aversion in riskless choice: A reference-dependent model. Q. J. Econ..

[30] Lane D. (2016). The assumption of sphericity in repeated-measures designs: What it means and what to do when it is violated. Quant. Methods Psychol..

[31] Boschini M., Falasconi L., Giordano C., Alboni F. (2018). Food waste in school canteens: A reference methodology for large-scale studies. J. Clean. Prod..

[32] Huijg J.A., Gebhardt W., Crone M.R., Dusseldorp E., Presseau J. (2014). Discriminant content validity of a theoretical domains framework questionnaire for use in implementation research. Implement. Sci.

[33] Fleuren M.A., Paulussen T.G., Van Dommelen P., Van Buuren S. (2014). Towards a measurement instrument for determinants of innovations. Int. J. Qual. Health Care.

[34] Evenhuis I.J., Vyth E.L., Veldhuis L., Jacobs S.M., Seidell J.C., Renders C.M. (2019). Implementation of guidelines for healthier canteens in Dutch Secondary Schools: A process evaluation. Int. J. Environ. Res. Public Health.

[35] My School (2020). Australian Curriculum, Assessment and Reporting Authority. https://myschool.edu.au/.

[36] Australian Bureau of Statistics (2018). Socio-Economic Indexes for Areas (SEIFA). 2016. Commonwealth of Austrliaa, Australian Burau of Statistics. https://www.abs.gov.au/ausstats/abs@.nsf/mf/2033.0.55.001.

[37] My School (2021). Australian Curriculum, Assessment and Reporting Authority. https://myschool.edu.au/.

[38] Rochira A., Tedesco D., Ubiali A., Fantini M.P., Gori D. (2020). School gardening activities aimed at obesity prevention improve body mass index and waist circumference parameters in school-aged children: A systematic review and meta-analysis. Child. Obes..

[39] Hendrie G.A., Anastasiou K., Brindal E., Wiggins B., Baird D.L., Johnson B.J., Bell L.K., Gardner C., Arguelles J.C., Kelaart A. (2022). Increasing children’s vegetable consumption: An evidence-based process to develop best practice guidelines.

[40] Finch M., Sutherland R., Harrison M., Collins C. (2006). Canteen purchasing practices of year 1–6 primary school children and association with SES and weight status. Aust. N. Z. J. Public Health.

[41] Delaney T., Sutherland R., Wyse R., Wolfenden L., Lecathelinais C., Janssen L., Reilly K., Wiggers J., Yoong S.L. (2019). A cross-sectional study of the nutritional quality of student canteen purchases from New South Wales primary-school canteens. Public Health Nutr..

