# Supplementary material for: Effectiveness of a Multi-Strategy Behavioral Intervention to Increase Vegetable Sales in Primary School Canteens: A Randomized Controlled Trial
Source: Nutrients. 2022 Oct 10;14(19):4218. doi: 10.3390/nu14194218 (PMC9573522; doi:10.3390/nu14194218)
Supplement: Supplementary file 1 [file nutrients-14-04218-s001.zip › nutrients-1932989-Supplementary materials.pdf]

nutrients

MDPI

Supplementary Materials

Table S1. Questionnaire statements that canteen managers responded to measuring their level of agreement with individual and environmental factors affecting multi-strategy intervention implementation.

Factor	Questionnaire statements[1]
Individual factors	
Role clarity and knowledge (3)	1. For me it was clear what activities I had to do to increase the vegetable offering in the canteen.<br>2. I had all the information I needed to increase the vegetable offering in the canteen.<br>3. I had enough knowledge to implement the information I received to increase the vegetable offering in the canteen.
Self-efficacy (3)	4. I found it easy to do my tasks regarding the implementation of the VegUP canteen study.<br>5. Other tasks were limiting my ability to perform my tasks for the VegUP canteen study.<br>6. I was confident that I could increase the vegetable offering in the canteen in various ways.
Attitude-beliefs (3)	7. I found it pleasant to do my tasks regarding the implementation of the VegUP canteen study.<br>8. I felt good, when I performed tasks regarding the VegUP canteen study.<br>9. I believe it is possible to increase the vegetable offering in canteens with foods that students like.
Attitude-Outcome expectations (4)	10. The effects of the VegUP canteen study are visible for me.<br>11. The VegUP canteen study led to a healthier offer in the canteen.<br>12. The VegUP canteen study will lead to students eating more vegetables.<br>13. Performing my tasks for the VegUP canteen study gave me satisfaction.
Routine (2)	14. For me it was easy to remember what I had to do to increase the vegetable offering in the canteen.<br>15. I checked regularly whether I performed all my tasks for the VegUP canteen study.
Motivation (1)	16. I am motivated to increase the vegetable offering in the canteen.
Skills (1)	17. My skills are sufficient to increase the vegetable offering in the canteen.
Professional Role (1)	18. In my opinion, it is my task to increase the vegetable offering in the canteen.
	Environmental factors
Need for support (3)	19. I needed more information to perform my tasks regarding the VegUP canteen study.<br>20. I needed more training to perform my tasks regarding the VegUP canteen study.<br>21. I needed more support to perform my tasks regarding the VegUP canteen study.
Innovation (2)	22. The recommended strategies can be adapted to the food policy vision of my school.<br>23. The recommended strategies benefit students of all backgrounds.
Perceived organizational support (4)	24. I had enough time to increase the vegetable offering in the canteen.<br>25. In my canteen, we have enough human resources to increase the vegetable offering.<br>26. In my canteen, we have enough space and equipment to increase the vegetable offering.<br>27. In my canteen, we had sufficient financial resources to increase the vegetable offering in the canteen.

[1] Rated using a five-point scale, from 1 = strongly disagree, to 5 = strongly agree.

Table S2. Individual and environmental factors affecting multi-strategy intervention implementation as perceived by the canteen managers (n = 8), shown as means and standard deviations (SD).

Factor	Factor mean (SD)[1]
Individual factors	
Role clarity and knowledge (3 items, α = 0.945)[2]	4.5 (0.5)
Self-efficacy (3 items, α = 0.447)	
I found it easy to do my tasks regarding the implementation of the VegUP canteen study.	4.4 (0.5)
Other tasks were limiting my ability to perform my tasks for the VegUP canteen study.	2.1 (1.2)
I was confident that I could increase the vegetable offering in the canteen in various ways.	3.8 (0.9)
Attitude (7 items, α = 0.935)	3.6 (0.7)
Routine (2 items, α = 0.949)	4.1 (1.0)
Motivation (1 item)	4.0 (0.8)
Skills (1 item)	4.0 (0.5)
Professional role (1 item)	3.6 (0.9)
Environmental factors	
Need for support (3 items, α = 1.000)	2.0 (0.8)
Innovation (2 items, α = 0.544)	
The recommended strategies can be adapted to the food policy vision of my school.	3.4 (0.9)
The recommended strategies benefit students of all backgrounds.	3.8 (0.7)
Perceived organizational support (4 items, α = 0.950)	4.0 (0.7)

[1] Measured with a five-point scale, from 1 = strongly disagree, to 5 = strongly agree. [2] Number of questions on which the factor was based and Cronbach's alpha. When Cronbach Alpha was >0.7 the ratings for these items were averaged per respondent for analysis, otherwise individual item questions were retained.

Table S3. Canteen manager evaluation (n=8) of seven specific intervention strategies on five acceptability factors[1], shown as means and standard deviations (SD).

Intervention strategy	Easy to implement	Children responded positive	Time / labour intensive	Wasteful	Sustainable in future
Selling Rainbow Dippers	4.1 (0.6)	1.5 (0.8)	2.4 (1.5)	3.8 (1.3)	1.1 (0.4)
Vegetable-containing items positioned higher in online menu	4.6 (0.8)	2.7 (1.5)	2.1 (1.7)	2.7 (1.7)	2.9 (1.3)
Increased vegetable content in existing hot meals	4.6 (0.5)	3.5 (1.5)	1.9 (1.1)	1.8 (1.2)	3.4 (1.6)
Default lettuce/cucumber in sandwiches	4.5 (0.8)	2.6 (1.4)	2.0 (1.2)	2.3 (1.3)	3.0 (1.6)
Default beetroot in burgers	4.4 (0.9)	1.8 (0.9)	1.6 (1.2)	2.0 (1.4)	2.6 (1.6)
Pizza muffin with tomato slice	4.5 (0.8)	3.6 (1.2)	1.5 (1.1)	2.1 (1.4)	3.1 (1.7)
Replaced fruit with vegetables in Bento Box	4.8 (0.5)	3.8 (1.4)	1.5 (1.1)	2.0 (0.9)	3.1 (1.5)

[1] Measured with a five-point scale, from 1 = strongly disagree, to 5 = strongly agree.
